# Health Risk, Income Effect, and the Stability of Farmers’ Poverty Alleviation in Deep Poverty Areas: A Case Study of S-County in Qinba Mountain Area

**DOI:** 10.3390/ijerph192316048

**Published:** 2022-11-30

**Authors:** Jie Song, Yaping Cai, Yahong Wang, Salim Khan

**Affiliations:** 1 School of Social and Political Science, University of Edinburg, Edinburgh EH8 9YL, UK; 2 School of Statistic Economic, International College, Zhengzhou University, Zhengzhou 450001, China; 3 School of Management, Zhengzhou University, Zhengzhou 450001, China; 4 Business School, Zhengzhou University, Zhengzhou 450001, China

**Keywords:** health risk, poor farmers, poverty alleviation, DID model, S-county

## Abstract

Health status and income level are both important factors in reducing poverty and accomplishing sustainable development in deep poverty areas of China. Therefore, the purpose of this study is to provide policy support for the sustainable poverty alleviation of farmers by analyzing the net effect of health risk on Farmers’ income poverty and its impact mechanism. Based on the data of more than 199,000 farmers, this study uses the Difference in Difference (DID) model to empirically analyze the effect of health-risk on farmers’ income poverty. The empirical findings obtained from DID model show that health risk has a significant and positive impact on income poverty, where the impact of disability is higher. Furthermore, the mechanism shows that the impact of health risks on income poverty is mainly influenced by farmers’ off-farm working choices and time. The heterogeneity analysis shows that the health risk significantly impacts non-vulnerable farmers’ poverty. With outdated healthcare facilities in poverty-stricken areas, people are more likely to fall into income poverty. Therefore, the study concludes that establishing an effective long-term mechanism of health risk prevention is essential to improving the endogenous development power of poor farmers and decreasing income poverty.

## 1. Introduction

Health risk and poverty are commonly believed to have a bi-directional association and causality. At the same time, many poverty-led diseases and viruses’ disproportionality badly affect impoverished people and accelerate the vicious cycle of extreme poverty because of declined production efficiency. It leads to long-run disability and disease [1,2,3,4,5]. Health risk is the main reason for extreme poverty for the underprivileged. Poor people from rural generally live in unhealthy and harmful atmospheres without clean drinking water, satisfactory sanitation, and decent housing facilities [6,7,8,9]. The low quality or less care in poor communities and the lack of proficient medical staff might further expedite the health-risk output [10]. In addition, health risk contributes to the production and reproduction of extreme poverty [11]. Expenditures on health risks most frequently contain a large proportion of poor farmers’ incomes that increase their financial burden [12,13]. Demand for health care and paying for it has become a distinguished cause of farmers’ poverty, while ineffective and poor healthcare quality intensifies the economic burden for poor households [14,15]. Farmers who live in deep poverty areas with less socioeconomic status have high health risks such as noncommunicable diseases (NCDs) [16,17]. Meanwhile, productive economic activities depend on human capital. Therefore, the reduction of poverty and eradication of health risk has become a top priority of researchers to ensure the United Nations (U.N.) 2030 Sustainable Development Goals (SDGs), for example, reduce poverty and ensure eradication of health risk by ensuring healthy live [18,19,20].

Alternatively, the impact of poverty generally disturbs the people’s living status, which affects social and economic status [21]. Economically impoverished households cannot handle the high health risk expenditures compared to richer households [22]. The impoverished mostly behave with low ability and less capable ways and suffer from disproportionate health risks, yet generally have less or no access to better health care [14,16]. As yet, many research studies have found and focused on the poverty-health nexus [23], driven by ill-health and poverty trap [24,25,26], the vicious cycle of poverty and ill-health [27,28], ecosystem poverty and interaction of health [29,30], and medical poverty trap [31]. Nevertheless, many research studies empirically investigated the association between poverty and ill health status, while few explored the connection between poverty and health-income-place.

In the past decades, China was enlisted as the world’s largest country with a higher poverty rate, while the healthcare system problem has attracted global attention. It has been involved in reducing health risks and poverty since 1974. Countless efforts have been made to reduce health risks by improving the healthcare system and implementing health poverty reduction projects in countryside China [32,33,34]. However, the countryside’s poverty problems remain serious and become more difficult to reduce by implementing traditional anti-poverty methods. Most past research studies inspected the association between consumption/income poverty in the literature, using morbidity, mortality, and nutrition as the key health status indicators [30]. In China, studies on alleviating poverty focus on households rather than individuals. At the same time, few measure the empirical relationship between ill health and poverty by focusing on impoverished people [35].

In 2013, about 69.9 million people in rural China lived below the poverty line of $376 (2300 CNY); 43% of them became poor because of ill-health status, while more than 13% were suffering from serious or chronic diseases. Though the total number of countryside poor due to ill health reduced from 29 million to 6 million at the beginning of 2019, the rate of poverty because of ill-health status remained at about 39%. Hence, it is important to recognize the health status of the rural poor in China and the mechanism of how health risk leads farmers into poverty [36]. To this end, the current research mainly focuses on the expenditure effect of health risk (the impact of health risk) on poor farmers from the perspective of consumption poverty and the effect of poverty reduction on the New Rural Cooperative Medical System (NCMS). There are a few studies on the income effect of health risk and the impact of it on the poverty status of farm households from the perspective of income. While the impact of the expenditure effect of health risk on the poverty status of farmers has been better controlled, a question that still needs attention is to what extent will the income effect of health risk influence the poverty status of farmers and their stability out of poverty? Are there any differences in the income poverty effects of different health risk shocks and the income poverty effects of different farm households exposed to health risk shocks? Does the intervention on the income effects of health risk shocks significantly improve the stability of farm households out of poverty? The research in this paper has some reference implications for risk coping and the stability out of the poverty of farm households in deep poverty areas.

## 2. Theoretical Background and Hypothesis

The Sustainable Livelihoods Approach (SLA) established the framework for international development. This approach was initiated from rapid rural appraisal, agricultural ecosystem analysis, agriculture system and participation, and applied anthropology [37,38,39]. Later, the SLA has been extensively utilized by local and central governments of developing regions and has become the vital model of small landholders (poor) farmers’ livelihood analysis [40]. In the fragilized situation, some livelihood capitals were strictly vulnerable, affecting poor farmers from deep-poverty areas. The SLA approach emphasizes how the poor community, household, or individual obtains basic capital (social, economic, and human capital), which plays a vital role and shows a significant innovation for empirical research on sustainable livelihood for poor farmers [9]. The previous studies adopted the SLA approach in their studies on poverty reduction. They suggested policy recommendations for policymakers to help farmers from deep-poverty areas move out of poverty and accomplish sustainable development [41,42].

It is noted that the SLA can be adopted to improve the management system of rural natural and non-natural resources, which greatly benefits poor people living in poverty-stricken areas [43]. Due to floods and droughts, health risks can increase in deep-poverty areas, negatively impacting farmers’ income. Economic vulnerability and income farmers’ income poverty have become the leading factor restricting China’s advancement and development in poverty-stricken areas [44]. Some researchers considered that higher non-farm income share was not always parallel with a higher viable pathway to attain sustainable livelihood in addition to non-farm employment [45]. In economically vulnerable conditions, investment decision-making is shaped by the psychological context of life. Therefore, the work of Zheng, Ma [45] documented that effective policies and programs related to poverty reduction should consider the phycological context of decision-making and poverty. Against the drawback, the above discussion, and theoretical framework, the study proposes a few research hypotheses as follows:

**Hypothesis 1:**  *Health risk has a homogeneous relationship with the income poverty of farmers in deep poverty areas.*

**Hypothesis 2:**  *Health risk positively affects the poverty status of farming households by influencing their choice of work and working hours.*

**Hypothesis 3:**  *There is heterogeneity in the effect of health risk on the income poverty status of farm households.*

Poor farmers from poverty-stricken areas face socioeconomic, environmental, and physical (health) risks and diseases, which are the main factors in their agricultural productivity and scale operation. These uncertainties and the livelihood of farmers can block the ways for poor farmers to escape extreme poverty [46,47]. Poor environmental mitigative policies in deep-poverty areas can increase environmental instability, leading to increased health risks. The general people and poor farmers from poverty-stricken areas are often the first victims of natural diseases due to climate change [48,49]. For instance, many previous empirical studies revealed that most of the poor people living in montunos backward areas are related to agriculture. They face a poor natural atmosphere, weak sanitation, lack of clean drinking and non-drinking water supply, and poor land conditions. According to the work of Shameem, Momtaz [50], the farmers living in poverty-stricken areas are more socially and economically vulnerable to natural disasters such as environmental risk. The main risk to farmers’ health is climate change, while the health risk determines their agriculture productivity and income level [51]. Income poverty is mainly caused by farmers’ lack of self-development, awareness of social participation, and health protection [52].

## 3. Materials and Methods

### 3.1. Method and Model Setting

How can the effect of health risk on income poverty be identified accurately? Besides health risks, many factors such as regional economic development, resources and environment, farmers’ development consciousness, and development capacity cause poor farmers to fall into or out of poverty [53]. How accurately identify the net effect of health risks on the farmers’ income poverty, that problem needs to be addressed in this paper. To this end, other factors that affected the farmers’ income poverty must be excluded. In this paper, we use the DID model to empirically analyze the effect of health–risks on farmers’ income poverty. It controls for unobservable fixed differences in characteristics between the treatment and control groups and the effect of unobservable aggregate factors that vary over time [54,55]. Whether or not one suffers a health risk depends not on the farmers themselves, and it can be considered random, i.e., the result of a natural experiment. The samples in this paper are drawn from deep-poverty counties, and the presence or absence of a health risk shock is the dividing line between the two groups of farmers. In addition, the infrastructural environment, market environment, natural environment, cultural environment, and support policies faced by the two groups of households are similar, while the criteria for identifying poor and non-poor households can exclude the biasness in estimation caused by the differences in these conditions. Therefore, we assume that the two groups of farmers have the same “time effect” trend. The model is set as follows:

(1) $$poverty_{it}=\beta_{0}+\beta_{1}healthrisk_{it}+\beta_{2}time_{it}+\beta_{3}time_{it}*healthrisk_{it}+\delta X_{it}+\varepsilon_{it}$$

where in Equation (1), $poverty_{it}$ is the dependent variable representing the poverty status of farm households with number *i* at time *t*. The poverty status refers to absolute poverty, which follows the Chinese rural poverty standard of the record-keeping card in China. When the answer to the question on farmers’ poverty status of “general farmers” *poverty_it_* = 0; otherwise, *poverty_it_* = 1. In this paper, the time dummy variable (*time_it_*), the health-risk dummy variable (*healthrisk_it_)* and its interaction term (*time_it_* * *healthrisk_it_)* are selected as the main independent variables. Among them, *time_it_* represents the time effect of farmers’ poverty status change from 2016 to 2018; *healthrisk_it_* is a dummy variable for whether a farmer with the number *i* is in the experimental group or the control group in year *t*. The coefficient indicates the effect of health risk when the farmers’ health status in that year having chronic diseases, disability or severe illness, *healthrisk_it_ =* 1. It indicates that the farmer is exposed to a health risk shock and is categorized as an experimental group. When the answer is (‘healthy’), *healthrisk_it_* = 0, indicating that the farmer is not exposed to a health risk shock and is classified as a control group. The interaction term between the two was used to measure the net effect of health risk on the poverty status of farm households, that is, the difference in the effect of health risk on the poverty status of farm households in the treatment and control groups throughout 2016–2018. *X_it_* represents control variables affecting the poverty status of farm households with the number *i* in year *t*, and ε denotes the random disturbance term for the poverty status of farm households numbered *i* at year *t*. The relevant variables are defined in Table 1.

### 3.2. Data Description

This paper mainly adopts the data of farm households in *County S*, part of China’s contiguous destitute area. It covers the main information related to the poverty of farm households in deep poverty areas, and the dataset contains a sample of 199,133 farm households from 2016 to 2018. The total population of County S is about 620,000. There were still about 44,000 people in poverty in 2018, of which 46.2% were poor due to disability and disease, 32.5% were poor due to lack of capital and technology accounted for 32.5%, poverty due to labor shortage accounted for 11.03%, and poverty due to schooling accounted for 3.97%. The data covered 16 townships in the county. Table 2 illustrates the descriptive statistics for each variable.

## 4. Results and Discussion

### 4.1. Analyzing the Impact of Health Risk on Farmers’ Income Poverty

In this study, the impact of health risks on the poverty status of farmers in deep-poverty areas was first tested by regression using the full sample. Afterward, model (1) is re-tested by introducing farmers suffering from major disease risk, disability risk, and long-term chronic disease as sub-samples and their poverty effects. It is important to clarify that some farmers in deep poverty areas may suffer from two or more health risks simultaneously or at different times, i.e., the same farmer may belong to different subsamples simultaneously. In this way, there may be a certain subsample whose poverty effects are overestimated, but this does not affect their ranking. All models in this section use robust standard errors for robust, and the specific regression results are shown in Table 3.

In model (1), DID is the interaction term between the real health risk and time, indicating the net effect of the overall health risk on the poverty status of farmers. The results indicate that health risk has a significant positive impact on the poverty status of farmers in deep poverty areas. After classifying health risk into serious illness, disability, and chronic disease, the DIDs in models (2) to (4) are the components of health risk, respectively, that is, the interaction term of suffering from serious illness, disability, and long-term chronic disease, and time, indicating the net effect of the risk of serious illness, disability, and long-term chronic disease on the poverty status of farmers. The results show that serious illness and disability positively impact farmers’ poverty, respectively, even at a 1% level of significance, which will lead to farmers’ poverty. In comparison, chronic diseases positively impact farmers’ poverty at a 10% significance level. Therefore, as a whole, the impact of health risks on farmers’ poverty has a positive impact, which verifies hypothesis 1. The findings obtained here are compatible with the work of Daghagh Yazd, Wheeler [56], and Danso-Abbeam, Ehiakpor [57].

### 4.2. Validity Analysis (Robustness Tests)

To enhance the robustness of the model and assess the poverty effect of health risk on farmers more accurately, the paper introduces control variables based on the basic DID model. According to Table 4, control variables such as whether there is a lack of labor (queziyuan1), natural disaster risk (disasterrisks), whether the household is covered by sickness insurance (sickinsurance), an education level (educ), working time, and age. Three combinations of control variables were selected, controlling for factors affecting the dependent variable from simple to complex (details in Table 4).

In DID model (1), there are only main variables. They are time, healthrisk, their interaction items, and dependent variables (farmers’ poverty status), and the set of control variables is empty. It can be seen that the DID parameter of the interaction term is estimated to be positive, and its *p*-value is less than 0.01 in the model (1). It indicates that health risk has a significant positive impact on the poverty status of farmers in deep poverty areas. The farmers who suffer from health risk impact in deep poverty areas are more likely to be poor than those who do not suffer from the health risk. The findings are consistent with the work of Chen and Cao [58] in the context of the Chinese economy and Pothisiri, Vicerra [59] in Thailand. To avoid problematic parameter estimates of standard errors, this study re-requested the estimation of bootstrapped standard errors, and the results did not differ from model 1. Models 2–4 add control variables for farmers’ vulnerability (vulnerability), labor shortage (labor), natural disaster risk (disasterrisks), major illness insurance (sickinsurance), working time outside(worktime), education level (educ), age (age), the average age of the adult population in rural households (meanage) as control variables. As can be seen from the results, adding these control variables did not change the significance level of the DID term. The significance level (*p*-values) of the parameter estimation of interaction item (β3) in the models were all less than 0.01, i.e., with the addition of the control variables, the model still had a positive effect on health risk at the 1% significance level.

### 4.3. Robustness Test Based on PSM-DID

Based on the results of the descriptive statistics, the median and mean values of farmers’ income in both the experimental and control groups increased between 2016 and 2018. Still, the control group has a significantly higher income level than the experimental group, suggesting that the DID model estimation is justified. The exact magnitude of the impact of health risk needs further validation. The parameter estimates may be biased if the overall population is regressed on whether or not they suffered a health risk shock as a dummy variable. The PSM-DID approach allows us to estimate the probability of a health risk for a household and then select a control group of households with a similar probability but without health risk to analyze the difference in their poverty status. Thus, further robustness checks are conducted using the PSM-DID method to overcome the estimation bias arising from systematic differences in the trends of poverty status between farmers exposed to health risk and other farmers. First, a logit regression was applied to the control variables utilizing a dummy variable for whether the household suffers from a health risk shock to obtain a propensity score. The household with the closest propensity score is the paired household with a health risk shock. By matching the propensity scores, the systematic error in the poverty status of different households is minimized, thus reducing the estimation bias of the standard DID method. Second, it is important to test whether the variables are significantly different between the experimental and control groups after matching, i.e., jointly supporting the hypothesis that if the variables are not significantly different, the model can be used directly for estimation.

The results show that the radius matching and nearest neighbor matching results are the same. The significance of the interaction term and the direction of influence in the estimation results of the PSM-DID model remain unchanged compared to the ordinary DID (the model fits better and has a larger influence coefficient), indicating that the estimation results are robust, i.e., health risk significantly increases the likelihood of farmers in deep poverty areas falling into poverty or maintaining poverty, and increase the likelihood of poor households staying in poverty as well as getting out of poverty. The estimated results of the PSM-DID model are not significantly different from those of the ordinary DID model, thus further validating the empirical findings of this paper.

## 5. Further Discussion

### 5.1. Heterogeneity Analysis

There may be heterogeneity in the impact of Health-Risk on the poverty status of farmers with individual characteristics. This part discusses the impact of possible heterogeneity factors on the net effect of health risk in detail. When other conditions are similar, comparing the effects of health risks on farmers with different characteristics, such as whether they are vulnerable, whether labor resources are short, traffic conditions, self-development motivation, working conditions, working time, and so on, the paper adds cross terms based on the basic DID model, as shown in Equation (2).

(2) $$poverty_{it}=\beta_{0}+\beta_{1}healthrisk_{it}+\beta_{2}time_{t}+\beta_{3}time_{t}\times healthrisk_{it}+\delta X_{it}\;+\beta_{4}healthrisk_{it}\times X_{it}+\beta_{5}time_{t}\times X_{it}+\beta_{6}time_{t}\times X_{it}\times healthrisk_{it}+\varepsilon_{it}$$

In Equation (2), *X_it_* represents the individual characteristics of farmers in deep poverty-stricken areas. The coefficient of *healthrisk_it_* × *X_it_* (*β*_4_) indicates that compared with the farmers without these features, the farmers with this feature are affected by the inherent differences between the treatment group and the control group. The coefficient of *time_t_ × X_it_* (*β*_5_) indicates that compared with the farmers without these features, the farmers with these features are affected by the unobservable factors that change with time. The coefficient of *time_t_ ×X_it_ × healthcare_it_* (*β*_6_) is the key point we focus on in this part, indicating the differences in the poverty status of farmers with different characteristics after the impact of health risk. The estimated results of the model are shown in.

In Table 5 (model 1), the coefficient of did_1, that is, health-risk * time * cuiruo is significantly negative (−0.1330) at 1%, indicating that compared with vulnerable farmers, the impact of health risk on the poverty status of non-vulnerable farmers is greater, that is, the impact of the same magnitude of health risk on non-vulnerable farmers is more obvious. The possible explanation is that China is in the key period of poverty alleviation from 2016 to 2018, and the vulnerable farmers themselves are in a state of poverty or on the edge of poverty. If they suffer from health risks at the same time, the government may give them more support. Therefore, the sign is negative in the case of both situations (vulnerable and suffering from health risks at the same time). (2) The coefficient of did_2, that is, health-risk *time *queziyuan2 is significantly negative (−0.0286) at 5%, indicating that the impact of health risk on the poverty status of farmers who are not lacking in the labor force is greater, but the impact on the poverty status of farmers who are lack of labor force is weaker. The possible explanation is similar to did_ 1. Because the farmers who lack a labor force are in the scope of policy support, their poverty status is less sensitive to the impact of a health risk than the farmers who are not lacking a labor force. (3) The coefficient of did_3, that is, health-risk *time *work, is significantly positive (0.0220) at 5%, indicating that health risk has a greater impact on the poverty status of migrant farmers in deep poverty areas, that is, the same range of health risk impact has a more significant positive impact on the poverty status of migrant farmers than that of non-migrant farmers. The findings of the current study support the empirical work of Mucci, Traversini [60].

### 5.2. Mechanism Test—Based on Mediating Model

Limited availability of the longer dataset for the analysis, this paper studies the short-term effects of health risk only, in which mediating model is used to identify the effect mechanism of health risk on farmers’ income poverty by introducing more variables. Suppose the influence of explanatory variable X on the explained variable Y becomes smaller after adding the mechanism variables. In that case, it is considered that part of the influence of X on Y is realized through the mechanism variables, and the path is called one of the influence mechanisms. If it does not change significantly after adding the influence factors (mechanism variables), the variable may not be one of the mechanisms. The core of the test is to check whether or to what extent the independent variable (health risk) affects the mediating variables and ultimately affects the dependent variable.

According to the existing research, health risk impacts the choice of whether to go out for work or not and the working hours of farmers, while both affect the income level of rural households and their income poverty status. Therefore, the core of the mechanism test is to verify whether health risk has a positive impact on the poverty status of rural households by influencing their migrant working choice and working time:

(i) Verify the influence of health risks on the working or non-working hours of farmers:
  (3) $$wugong_{it}\left(workingtime_{it}\right)=b_{0}+b_{1}DID+\sum_{i=1}^{n}a_{j}X_{it}+\varepsilon_{it}$$
  In Equation (3), *work_it_* represents the impact of health risks on farmers’ choice of whether to go out for work or working time. *workingtime_it,_* represents the time farmers go out to work, expressed by the number of months that farmers go out to work in a year. The expected sign is negative, which indicates that health risk has a certain negative impact on farmers’ working time, and the income effect affects farmers’ poverty status and poverty alleviation stability. *DID* represent the net effect of health risk; if the coefficient of DID (*b_1_*) is significant, it indicates that the health risk does affect the choice of whether to work or not and the time of working; that is, there is the expected impact in the theoretical analysis framework, so it can be considered that there is a corresponding impact mechanism. If it is not significant, the corresponding mechanism analysis is excluded. *X_it_* represents the control variables, mainly including the vulnerability of farmers themselves, education, age, etc.; $\varepsilon_{it}$ represents disturbance item.
(ii) Verify the poverty effect of migrant choice and working time
  (4) $$poverty_{it}=b_{0}+b_{1}DID+\sum_{i=1}^{n}a_{j}X_{it}+\varepsilon_{it}$$
  In Equation (4), $poverty_{it}$ indicates the poverty status of farmers; other variables are the same as in model 3. If the coefficient is significant, it indicates that the health risk impact affects farmers’ poverty status. Whether the impact is realized through farmers’ choice of work and working time needs to be further verified.
(iii) The DID term, migrant choice (or working time) are put into the regression equation at the same time
  (5) $$poverty_{it}=b_{0}+b_{1}DID+\beta_{1}work_{it}\left(workgtime_{it}\right)+\sum_{i=1}^{n}a_{j}X_{it}+\varepsilon_{it}$$
  Equation (5) is the poverty effect model of health risk, which is used to evaluate the impact of health risk on the poverty status of farmers in deep poverty areas. If the coefficient of DID (*b*_1_) is not significant or still significant, but the absolute value of the coefficient decreases after adding the mechanism variables, it indicates that part of the impact of health risk on the poverty status of farmers is realized through the mechanism variables, that is, this is one of the impact paths of health risk on the poverty status of farmers. If the coefficient of DID (*b*_1_) does not change significantly after adding the mechanism variable, then the variable may not be one of the mechanism variables. The estimated results are obtained using the S County data, as shown in Table 6.

Model 1 in Table 6 is the first step to test. The results show that the net effect of health risk (DID item) has a significant positive impact on the poverty status of farmers at a 1% level. Still, the specific impact mechanism needs to be further tested. The results of the second step test (models 2 and 3) show that the coefficient of DID (b_1_) of the two models is significantly negative at 1% level, which indicates that the health risk has a significant negative effect on farmers’ choice of whether to go out to work and working time. The results of the third step test (models 4 and 5) show that the coefficient of DID (*b*_1_) is all smaller than that of the models without adding the mechanism(mediating) variables. However, both of them are still statistically significant, even at 1% level. The results show that the mediating effect of the two mediating variables is significant, and mediating variables explain some impact of health risk; that is, health risk impact does have a positive impact on farmers’ poverty status by decreasing farmers’ choice of whether to go out to work and working time, which verifies hypothesis 2 and hypothesis 3.

## 6. Conclusions and Policy Recommendation

The main results of the study can be summarized in the following four ways:

i Health risk has a significant positive impact on the income poverty of farmers in deep poverty areas. Limited availability of a longer dataset, this study only empirically analyzes the short-term effects of health risk shock. We confirm the robustness of the estimate by adding control variables and using the PSM-DID model.
ii The analysis of the impact mechanism of health risk on farmers’ income poverty shows that under the condition of effective control of its expenditure effect, health risk can also affect farmers’ poverty status through income effect; that is, it can affect farmers’ income by influencing farmers’ Off-farm working choice and working time.
iii The heterogeneity analysis of health risk impact shows that the impact of serious diseases, long-term chronic diseases, and disability on farmers’ income poverty are all statistically significant and positive. Moreover, the shock of disability risk has the greatest impact on farmers’ income poverty, followed by the impact of major illness, and long-term chronic diseases rank third.
iv The heterogeneity analysis of farmers’ characteristics shows that the impact of health risk has a more significant impact on income poverty of non-vulnerable farmers, farmers who do not lack a labor force, those who choose to work outside, and farmers who work longer. That is, under the same impact level of health risk, non-vulnerable farmers are more affected than the vulnerable farmer, farmers who do not lack a labor force are more affected than those who lack a labor force, and those who choose to work outside are more affected than those who do not go out for work.

Therefore, it can be suggested that more attention should be paid to the income effect of health risk, especially the poverty status of farmers who cannot enjoy the specific poverty alleviation policy (who are not fragile, who are not lacking labor, those who choose to go out for work and those with longer migrant-working time) to improve the stability of poverty alleviation of farmers in deep poverty areas, especially in the post-poverty alleviation. In addition, the central government and local concern authorities of the selected deep-poverty areas should increase the socioeconomic resources of poor farmers through welfare and poverty alleviation programs. The policymakers should provide more information about the prevailing market structure to poor farmers in addition to capital support. The encouragement and support from the government sector will increase the ability of poor farmers to establish social resources and develop the market independently.

At the same time, the current measures, such as New Rural Cooperative Medical System (NCMS) and the major disease insurance scheme, are mainly focused on reducing the medical expenditure of farmers (the expenditure effect of health risk), which has limited impact on the income poverty of farmers suffering from health risk in deep poverty areas. While health risks attack them, the labor force and migrant working hours would be reduced, and their income would be reduced. It is suggested that more attention should be paid to the income effect of health risks. To protect farmers from poverty again caused by health risk attacks, particular attention should be paid to the income reduction of farmers who have now been lifted out of poverty and do not benefit from pro-poor policies. Finally, we should improve the health awareness of farmers and reduce the impact of health risks to ensure the stability and sustainability of poverty alleviation of farmers in deep poverty areas.

In addition, the novelty and main contribution of the study are as follows:

i This paper studies the impact mechanism of health risks on farmers’ income by analyzing the health risk and income poverty. It provides a new theoretical perspective for policymakers in deep poverty areas to alleviate income poverty.
ii It depicts the “instability” and “heterogeneity” of poverty alleviation of farmers in deep poverty-stricken areas and explains health risks in the context.
iii In the empirical method, DID model is used to analyze the impact and heterogeneity of health risk shocks on farmers’ income poverty and identify the relationship between the net effect of risk shocks and rural household income poverty.
iv This paper emphasizes the problem of missing assistance caused by the mismatch of poverty alleviation resources, that is, the missing assistance caused by the biased coverage of assistance objects; discusses the impact of the optimal allocation of limited poverty alleviation resources on the stable and sustainable poverty alleviation of farmers in deep poverty-stricken areas; provides the theoretical basis and practical guidance for the optimization of precise poverty alleviation strategies in deep poverty-stricken areas; and improves the efficiency of poverty alleviation by optimizing the structure of limited poverty alleviation resources.

Moreover, the health risk is among the most common and influential risks farmers face in deep poverty areas. New Rural Cooperative Medical System (NCMS) and the major disease insurance scheme focus on the relationship between health risk and farmers’ medical expenditure, which has greatly reduced the increase of expenditure caused by health risk, especially the major illness insurance reduced the impact of current medical expenditure. However, the income effect of health risks on farmers’ poverty is still insufficient. The results show that health risks negatively impact the poverty status of farmers who are not fragile, who is not short of labor, and those who choose to go out to work, which is more likely to aggravate their poverty status or bring them back to poverty. To shake off poverty in deep poverty-stricken areas, we should pay attention to the impact of risk, especially the impact of health risk on the stability of farmers’ poverty alleviation, which is essentially the income effect of health risk. Therefore, the accurate poverty alleviation policy needs to treat different types of farmers differently. Not only should we pay attention to the impact of health risks on farmers’ expenditure, but we also pay attention to its impact on Farmers’ income. It is suggested that the income guarantee of health risk be improved and the time opportunity cost during treatment and recovery should be reduced.

Although the outcomes of the current study are significant for the Chinese economy, however, not without limitations that should be considered and extended for future research; in this regard, a health risk may not only have a significant positive impact on the current poverty status of rural households through income and expenditure effects but may also affect their long-term income ability and income level by affecting their capacity of capital accumulation. When farmers suffer health risks that may ultimately result in increased expenditure, incomes squeezed, or both threatened, they are more likely to use limited resources for current consumption and reduce investments. Indeed, health risks, expenditure increase or incomes squeezed or both, insufficient investment in investment (including productive and non-productive investment), insufficient income raising capacity and opportunities, and lower income may build up a vicious circle, even resulting in the intergenerational transmission of this vicious circle, which makes it more likely that farmers will fall into poverty in a certain period in the future. But due to the data accessibility restrictions, the long-term impact of health risks on farmers’ stability of poverty alleviation in deep poverty areas is not verified in the paper.

## Figures and Tables

**Table 1:** Definition of variables.

Dependent Variables	Variables	Variable Definitions
	Poverty	Poverty is 1, Non-Poverty Is 0
Independent variables	healthrisk	1 for the experimental group and 0 for the control group
	time	0 in 2016 and 1 after 2016
	DID	Time × healthrisk indicates the net effect of health risk
Controlling variables	vulnerability	Vulnerable is 1, not vulnerable is 0
	labor	1 for labor shortage, 0 for no shortage
	disaster	Exposure to natural disaster risk shocks (1 = yes, 0 = no)
	Sickinsurance	Whether to participate in major medical insurance (1 = yes, 0 = no)
	Work	Whether or not you choose to work outside the home (1 = yes, 0 = no)
	worktime	Duration of work outside (months)
	Educ	Level of education (1 = illiterate or semi-literate, 2 = primary school, 3 = junior high school, 4 = high school, 5 = college and above)
	age	age

**Table 2:** Descriptive statistics of variables (2016–2018).

Variable	Mean	Std. Dev.	Min	Max	Obs
poverty	0.605	0.489	0	1	199133
healthrisk	0.202	0.402	0	1	199133
income	8.119	0.493	3.714	11.194	199133
vulnerability	0.825	0.38	0	1	199133
healthrisk1	0.014	0.117	0	1	199133
healthrisk2	0.059	0.235	0	1	199133
healthrisk3	0.116	0.321	0	1	199133
technicalrisks	0.199	0.399	0	1	199133
diseaserisks	0.257	0.437	0	1	199133
Disabilityrisks	0.076	0.266	0	1	199133
technicalrisks	0.199	0.399	0	1	199133
financialrisks	0.283	0.451	0	1	199133
labor	0.067	0.251	0	1	199133
sickinsurance	0.996	00.06	0	1	199133
worktime	1.372	2.603	0	12	199133
educ	2.469	0.904	0	5	199133
work	0.278	0.448	0	1	199133
age	40.635	22.106	1	103	199133

**Table 3:** Impact of health risk on farmers’ poverty status in deep poverty areas.

Variables	(1)	(2)	(3)	(4)
time	−0.170 *** (0.00)	−0.156 *** (0.00)	−0.161 *** (0.00)	−0.159 *** (0.00)
healthrisk	0.215 *** (0.00)			
DID	0.080 *** (0.01)	0.112 *** (0.02)	0.105 *** (0.01)	0.042 *** (0.01)
healthrisk1		0.188 *** (0.01)		
healthrisk2			0.213 *** (0.01)	
	
healthrisk3				0.166 *** (0.01)
_cons	0.653 *** (0.00)	0.694 *** (0.00)	0.685 *** (0.00)	0.678 *** (0.00)
R2	0.071	0.028	0.042	0.040
N	199133	199133	199133	199133

Note: Standard errors in parentheses *** *p* < 0.01.

**Table 4:** Estimation results of the DID model.

	(1)	(2)	(3)	(4)
time	−0.170 *** (0.00)	−0.102 *** (0.00)	−0.102 *** (0.00)	−0.099 *** (0.00)
healthrisk	0.215 *** (0.00)	0.215 *** (0.00)	0.215 *** (0.00)	0.171 *** (0.00)
DID	0.080 *** (0.00)	0.065 *** (0.01)	0.065 *** (0.01)	0.062 *** (0.01)
vulnerability		0.348 *** (0.00)	0.347 *** (0.00)	0.335 *** (0.00)
labor		0.123 *** (0.00)	0.123 *** (0.00)	0.105 *** (0.00)
disasterrisks			0.147 *** (0.02)	0.142 *** (0.02)
sickinsurance			−0.252 *** (0.02)	−0.245 *** (0.02)
worktime				−0.012 *** (0.00)
educ				−0.036 *** (0.00)
age				0.00 1*** (0.00)
_cons	0.653 *** (0.00)	0.319 *** (0.00)	0.570 *** (0.02)	0.664 *** (0.02)
R2	0.071	0.145	0.146	0.155
N	199133	199133	199133	199133

Standard errors in parentheses, *** *p* < 0.01.

**Table 5:** Heterogeneous impact of health risk on the poverty status of farmers with different individual characteristics.

Variables	(1)	(2)	(3)
	Poverty	Poverty	Poverty
did	0.3119 *** (0.0069)	0.2100 *** (0.0036)	0.2055 *** (0.0037)
did_1	−0.1330 *** (0.0076)		
vulnerability	0.3243 *** (0.0032)	0.3051 *** (0.0030)	0.3051 *** (0.0030)
labor	0.1086 *** (0.0040)	0.1124 *** (0.0043)	0.1091 *** (0.0040)
disasterrisks	0.1193 *** (0.0161)	0.1207 *** (0.0161)	0.1205 *** (0.0161)
sickinsurance	−0.2057 *** (0.0169)	−0.2052 *** (0.0169)	−0.2055 *** (0.0169)
worktime	−0.0135 *** (0.0004)	−0.0137 *** (0.0004)	−0.0139 *** (0.0004)
educ	−0.0387 *** (0.0012)	−0.0388 *** (0.0012)	−0.0388 *** (0.0012)
age	0.0012 *** (0.0000)	0.0012 *** (0.0000)	0.0012 *** (0.0000)
did_2		−0.0286 ** (0.0127)	
did_3			0.0220 ** (0.0097)
_cons	0.6535 *** (0.0175)	0.6715 *** (0.0175)	0.6718 *** (0.0175)
R2	0.1682	0.1670	0.1670
N	199133	199133	199133

Standard errors in parentheses *** *p* < 0.01, ** *p* < 0.05.

**Table 6:** Mechanism test.

	(1)	(2)	(3)	(4)	(5)
	Poverty	Work	Workingtime	Poverty	Poverty
DID	0.866 *** (0.02)	−1.449 *** (0.02)	−1.076 *** (0.02)	0.824 *** (0.02)	0.781 *** (0.02)
vulnerability	1.724 *** (0.01)	−0.433 *** (0.01)	−0.906 *** (0.01)	1.711 *** (0.01)	1.676 *** (0.01)
educ	−0.255 *** (0.01)	0.623 *** (0.01)	0.605 *** (0.01)	−0.235 *** (0.01)	−0.212 *** (0.01)
age	0.007 *** (0.00)	0.016 *** (0.00)	0.011 *** (0.00)	0.008 *** (0.00)	0.008 *** (0.00)
work				−0.187 *** (0.01)	
worktime					−0.074 *** (0.00)
_cons	−0.736 *** (0.02)	−2.736 *** (0.03)	0.303 *** (0.02)	−0.741 *** (0.02)	−0.731 ***
R2 (_p)	0.096	0.083	0.087	0.097	0.102
N	199133	199133	199133	199133	199133

*t* statistics in parentheses *** *p* < 0.01.

## Data Availability

General correspondence and requests for source data and materials should be addressed to Wang Yahong. Requests for access to data should be addressed to wyahong2009@zzu.edu.cn.
